# Dataset of high-throughput ligand screening against the RNA Packaging Signals regulating Hepatitis B Virus nucleocapsid formation

**DOI:** 10.1016/j.dib.2022.108206

**Published:** 2022-04-26

**Authors:** Fardokht Abulwerdi, Farzad Fatehi, Iain W. Manfield, Stuart F.J. Le Grice, John S. Schneekloth, Reidun Twarock, Peter G. Stockley, Nikesh Patel

**Affiliations:** a Center for Cancer Research, National Cancer Institute, 21702-1201, Frederick, MD, USA; b Astbury Centre for Structural Molecular Biology, University of Leeds, Leeds LS2 9JT, UK; c Department of Mathematics, University of York, York, YO10 5DD, UK; d York Cross-disciplinary Centre for Systems Analysis, University of York, York, YO10 5GE, UK; e Department of Biology, University of York, York, YO10 5DD, UK

**Keywords:** Hepatitis B Virus nucleocapsid assembly, RNA Packaging Signal-mediated virus assembly, High-throughput screening, Assembly inhibitors

## Abstract

Multiple ssRNA viruses which infect bacteria, plants or humans use RNA Packaging Signal (PS)-mediated regulation during assembly to package their genomes faithfully and efficiently. PSs typically comprise short nucleotide recognition motifs, most often presented in the unpaired region of RNA stem-loops, and often bind their cognate coat proteins (CPs) with nanomolar affinity. PSs identified to date are resilient in the face of the typical error prone replication of their virus-coded polymerases, making them potential drug targets. An immobilised array of small molecular weight, drug-like compounds was panned against a fluorescently-labelled oligonucleotide encompassing the most conserved Hepatitis B Virus (HBV) PS, PS1, known to be a major determinant in nucleocapsid formation. This identified > 70 compounds that bind PS1 uniquely in the array. The commercially available 66 of these were tested for their potential effect(s) on HBV nucleocapsid-like particle (NCP) assembly *in vitro*, which identified potent assembly inhibitors. Here, we describe a high-throughput screen for such effects using employing fluorescence anisotropy in a 96-well microplate format. HBV genomic RNAs (gRNA) and short oligonucleotides encompassing PS1 were 5′ labelled with an Alexa Fluor 488 dye. Excess (with respect to stoichiometric *T* = 4 NCP formation) HBV core protein (Cp) dimers were titrated robotically into solutions containing each of these RNAs stepwise, using a Biomek 4000 liquid handling robot. The anisotropy values of these mixtures were monitored using a POLARstar microplate reader. NCP-like structures were challenged with RNase A to identify reactions that did not result in complete NCP formation. The results imply that ∼50% of the compounds prevent complete NCP formation, highlighting both PS-meditated assembly and the PS-binding compounds as potential directly-acting anti-virals with a novel molecular target. Importantly, this method allows high-throughput *in vitro* screening for assembly inhibitors in this major human pathogen.

## Specifications Table

**Table:** 

Subject	*Virology*
Specific subject area	PS binding compounds were identified [2] using PS1 from HBV gRNA as a target [1]. Their effect on NCP assembly were assessed in an anisotropy based screen in microplate format.
Type of data	TablesDataFigure
How the data were acquired	Anisotropy measurements were performed in a 96-well microplate using a microplate reader (plate reader - POLARstar Omega, BMG Labtech, plate – Greiner Bio-One, product no. 655900). Fluorescently-labelled RNA substrates and HBV Cp aliquots were titrated into the 96-well plate using a Biomek 4000 automated liquid handler robot (Beckmann Coulter).Anisotropy values were recorded using the Omega software (BMG Labtech), and subsequent analysis performed using the MARS software suite (BMG Labtech)
Data format	Analysed dataFigure
Description of data collection	Data were collected using the Omega software (BMG Labtech). Plates containing gRNA/compound/Cp or PS1 oligo/compound/Cp mixes were agitated for 30s prior to a 0.3 s settling time, and readings were taken using 200 flashes per well. Filter settings were set to excite each well at 485 nm, recording the emission at 520 nm. Prior to each assay, the gain was adjusted for each RNA substrate prior to Cp dimer titration.
Data source location	Institution: University of LeedsCity/Town/Region: LeedsCountry: United Kingdom
Data accessibility	*Raw data available:*https://data.mendeley.com/datasets/83tynj2x8z/3
Related research article	Nikesh Patel*, Fardokht Abulwerdi, Farzad Fatehi, Iain Manfield, Stuart Le Grice, John S. Schneekloth, Jr., Reidun Twarock & Peter G. Stockley* Dysregulation of Hepatitis B Virus Nucleocapsid Assembly *in vitro* by RNA-binding Small Ligands, J. Mol Biol. Under revision.*joint corresponding authors

## Value of the Data

• These data demonstrate a high-throughput method for assaying complete nucleocapsid assembly around fluorescently tagged RNA substrates.
• We have identified 30 PS1 binding compounds [2], which ablate the ability to form HBV NCPs.
• The PS-mediated assembly regulation mechanism occurs widely across many ssRNA viral families. The PS-ligand assay described herein provides a novel *in vitro* tool for identifying virus assembly inhibitors.

## 1 Data Description

Table 1: Cp dimer titrations used in NCP assembly assays.

**Table 1:** Cp dimer titrations used in NCP assembly assays.

Titration	Cumulative Cp Concentration (nM)	Cp Dimer Stock (nM)	gRNA (nM)	PS1 oligo (nM)
1	1	100	1.09	16.35
2	10	1000	1.08	16.2
3	25	2500	1.07	16.05
4	75	7500	1.06	15.9
5	120	12000	1.05	15.75
6	240	12000	1.04	15.6
7	480	24000	1.03	15.45
8	720	24000	1.02	15.3
9	960	24000	1.01	15.15
10	1200	24000	1	15

Left to right – titration number, the cumulative Cp dimer concentration after titration, the Cp dimer stock used and the resultant RNA concentration in each well after said titration.

Left to right – titration number, the cumulative Cp dimer concentration after titration, the Cp dimer stock used and the resultant RNA concentration in each well after said titration.

**Depository data:**

**Fluorescence Anistropy.xlsx**

Left to right; plates 1 to 3 – the compound used, and the associated raw anisotropy value.

**Normalised anisotropy Changes.xlsx**

Left to right; plates 1 to 3 – the compound used, and the normalised anisotropy value (= Raw anisotropy value/Raw anisotropy value of full untreated NCP).

**Averages and errors.xlsx**

Left to right – the compound used, and the average normalised anisotropy value of the triplicate plates and the associated standard error of the mean.

**Fig. 1:**
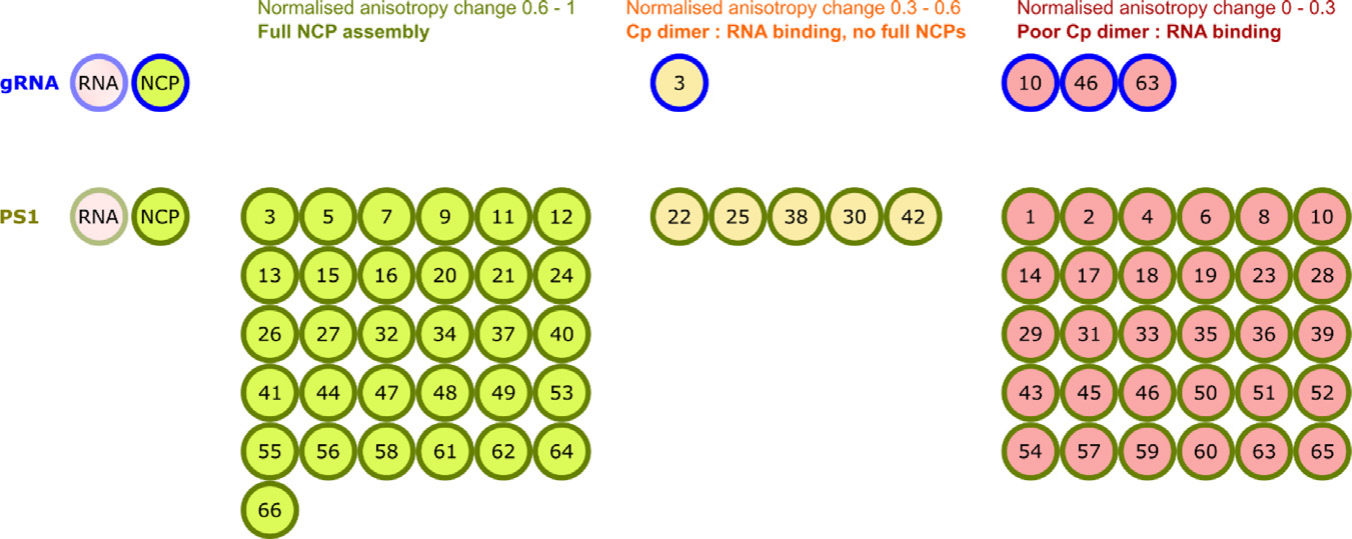
Summary of plate-based assembly assays. Heat annealed 1 nM gRNA (top) or 15 nM PS1 (bottom) oligonucleotide, [1]) were dispensed into a 96-well plate (blue, green outlines respectively). 10 µM of individual compounds (1-66) were added to wells, as labelled. 1.2 µM Cp dimer was titrated as described in Table 1 and any assembled material then challenged with 1 µM RNase A. The final normalised fluorescence anisotropy was calculated, and control reactions ± Cp used to benchmark values indicative of complete NCP (1) or degraded RNA (0). The anisotropy change was used to divide the results into three ranges, displayed here by the colour fill in each well. Left to right: *light green* 0.6-1, suggestive of efficient NCP assembly; *light yellow* = 0.3-0.6, suggestive of some Cp dimer: RNA binding; and *light red* = 0-0.3, suggestive of poor Cp dimer: RNA binding. Assay was performed in triplicate, and the average anisotropy change shown with associated standard error of the mean available in Depository data: averages and errors.xlsx, for PS1 and gRNA reassemblies, respectively.

Fig. 1. Summary of plate-based assembly assays.

Anisotropy change is displayed here by the colour fill in each well. Left to right: *light green* 0.6-1, suggestive of efficient NCP assembly; *light yellow* = 0.3–0.6, suggestive of some Cp dimer: RNA binding; and *light red* = 0-0.3, suggestive of poor Cp dimer: RNA binding. Assay was performed in triplicate, and the average anisotropy change shown with associated standard error of the mean available in Depository data: averages and errors.xlsx, for PS1 and gRNA reassemblies, respectively.

## 2 Experimental Design, Materials and Methods

### 2.1 Preparation of RNA assembly substrates

A pUC57 plasmid encoding for the gRNA substrate positioned between a 5´ T7 promotor sequence, and 3´ *HindIII* restriction site was purchased from Genscript. gRNA was transcribed from *HindIII*-linearised plasmid using a T7 polymerase HiScribe kit (New England Biolabs), supplementing the reaction with 5´ amino-GMP (Jena Bioscience), according to the manufacturer's protocol. The PS1 RNA oligonucleotide [2] was purchased from IDT with a 5´ amino group. Amino-labelled RNA substrates were prepared using Alexa Fluor 488 SDP ester (Thermo Fisher) in the presence of 100 mM sodium borate buffer, at room temperature for 4 h. Labelled PS1 was gel purified, and its’ integrity confirmed using a 1% w/v formaldehyde agarose gel [2].

### 2.2 Preparation of Cp dimers

Cp was expressed in BL21 (DE3) competent *E. coli* (New England Biolabs) from a pET28b plasmid containing the Cp gene [1]. Upon expression, Cp forms dimers, which in turn assemble into NCPs. Upon purification, these NCPs are dissociated, and the dimers subsequently purified, by dialysis into a buffer containing 50 mM Tris-HCl pH 9.5, 1.5 M GuHCl, 0.5 mM LiCl and 5 mM DTT, and subsequent size exclusion chromatography using a Superose 6 increase (Sigma Aldrich) column attached to an ӒKTA Pure system (Cytiva) [1], [3].

### 2.3 NCP assembly assays

gRNA and PS1 oligonucleotides were heat-annealed by heating to 70 °C, cooling slowly to room temperature in a buffer containing 10 mM MES pH 7.0, 25 mM NaCl and 1 mM DTT. RNA substrates were then diluted to working concentrations of 1.1 / 16.5 nM respectively in a buffer containing 25 mM HEPES pH 7.5, 250 mM NaCl and 5 mM DTT.

178 µL of RNA substrate was titrated using a Biomek 4000 liquid handling robot (Beckmann Coulter) into the wells of a 96 well plate (Greiner Bio-One, product no. 655900) and allowed to equilibrate at room temperature for 30 mins. 2 µL DMSO ± 10 µM compound 1 - 66 were added and a further equilibration step performed.

Purified Cp dimer was then titrated using the Biomek 4000 liquid handling robot stepwise into the RNA substrates as detailed in Table 1, up to a ratio of 1:1200 (RNA:Cp dimer). 1 µM RNase A was added when assembly reactions were complete.

Fluorescence anistropy was monitored throughout using a POLARstar Omega plate reader (BMG Labtech) and normalised with respect to control reactions in the absence of compound ± Cp dimer (Full NCP = 1, degraded RNA = 0).

## CRediT Author Statement

**Fardokht Abulwerdi:** Initial small molecule screen against PS1; **Farzad Fatehi:** *In silico* modelling of compound effects *in vivo*; **Iain Manfield:** Compound:RNA binding affinity methodology; **Stuart Le Grice, John S. Schneekloth Jr.:** SMM methodology, supervision and editing; **Reidun Twarock:** Conceptualization and supervision; **Peter G. Stockley:** Conceptualization, supervision and editing; **Nikesh Patel:** Investigation, formal analysis, conceptualization, fluorescence anisotropy methodology, writing and editing.

## Declaration of Competing Interest

The authors declare that they have no known competing financial interests or personal relationships that could have appeared to influence the work reported in this paper.

## Data Availability

Dataset Of High-Throughput Ligand Screening Against the RNA Packaging Signals Regulating Hepatitis B Virus Nucleocapsid Formation (Original data) (Mendeley Data).
